# Premature myocardial infarction presenting with acute pulmonary embolism: a case report

**DOI:** 10.4076/1752-1947-3-8786

**Published:** 2009-08-27

**Authors:** Seerapani Gopaluni, Aadil Shaukat, Martin Gray, Roshni Gokhale, M Al-Bustami

**Affiliations:** 1 Department of Cardiology, Cardiac Suite, Lister Hospital, Stevenage, SG1 4AB, UK

## Abstract

**Introduction:**

Myocardial infarction and coronary artery disease in people under 40 years of age are relatively uncommon. To establish a diagnosis, physicians need a high degree of clinical suspicion.

**Case presentation:**

We report the case of a 33-year-old Caucasian man presenting with classical signs and symptoms of acute pulmonary embolism. Subsequent investigations interestingly revealed right ventricular mural thrombus with no obvious underlying pathology triggering it. On cardiac magnetic resonance imaging, he was found to have a right ventricular infarct with secondary thrombus formation. Coronary angiography confirmed ostial occlusion of the right coronary artery.

**Conclusion:**

This is an unusual case of premature myocardial infarction presenting primarily with secondary complications.

## Introduction

Coronary artery disease and myocardial infarction occur more frequently in patients over the age of 40 years. However, they can affect patients younger than 40 years and clinicians need a very high degree of clinical suspicion to establish a diagnosis in this group. In this case report, we illustrate the occurrence of premature myocardial infarction in a 33-year-old man, who presented with symptoms from secondary complications.

## Case presentation

A 33-year-old Caucasian man presented to casualty with sudden onset of severe pleuritic chest pain associated with haemoptysis. He suffered from progressive dyspnoea and had felt generally unwell for a month before this presentation. He had received three courses of antibiotics from his general practitioner for a possible chest infection, to no effect.

His past medical history was of Ramstedt's pylorotomy for congenital pyloric stenosis. He had an 18 pack-year history of smoking and denied any use of recreational drugs. There were no other risk factors for cardiovascular disease.

On presentation, he was tachycardic at 120 beats per minute, and tachypnoeic at 36 breaths per minute. Pulse oximetry revealed oxygen saturation of 90% in room air. He was haemodynamically stable and clinical examination was unremarkable.

Blood investigations showed a haemoglobin of 13.9 g/dL, troponin-I of <0.04 ng/mL, creatine kinase of 17 U/L and D-dimer of 800. His electrocardiogram (ECG) showed right ventricular strain. Chest X-ray revealed ill-defined patchy shadowing in the left middle zone.

**Figure 1:**
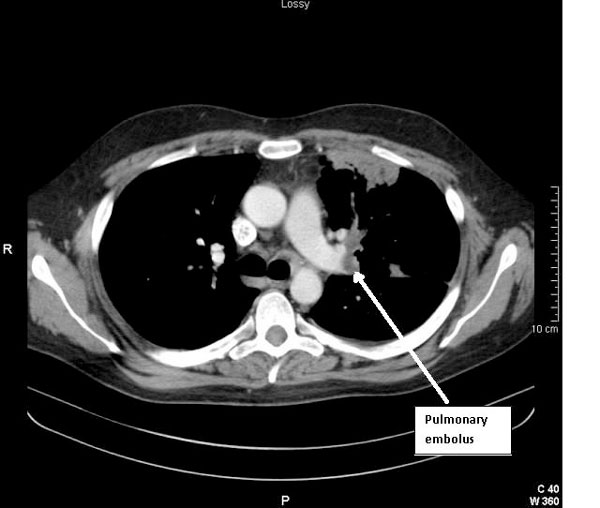
**Computed tomography image showing pulmonary embolism**.

**Figure 2:**
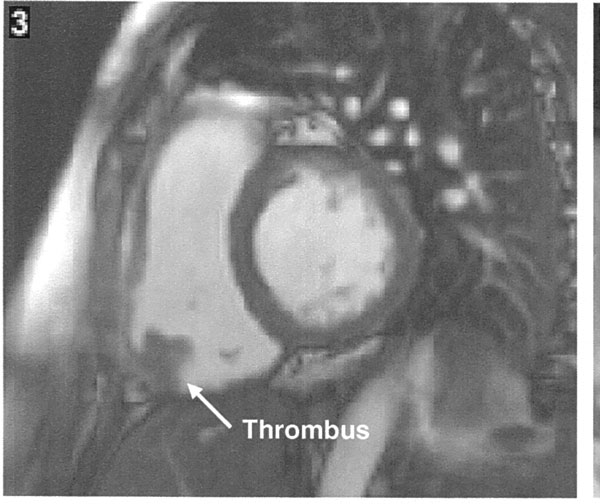
**Cardiac magnetic resonance imaging showing the thrombus attached to the right ventricle**.

Based on his classical clinical history and a raised D-dimer, a computed tomography pulmonary angiogram (CTPA) was arranged to investigate for possible pulmonary embolism. This showed filling defects in the left upper and lower lobe pulmonary arteries and several areas of parenchymal infarction, consistent with significant bilateral pulmonary emboli (Figure 1). Interestingly, it also revealed a mass in the right ventricle (RV), the nature of which was thought to be either a thrombus or a tumour (Figure 2).

An echocardiogram (ECHO), though suboptimal, demonstrated a normal functioning left ventricle with no regional wall motion abnormalities. The RV was mildly dilated but had normal systolic function. The mass seen on computed tomography (CT) was confirmed on ECHO, and was seen attached to the base of the lateral wall of the RV, with features suggestive of a thrombus.

**Figure 3:**
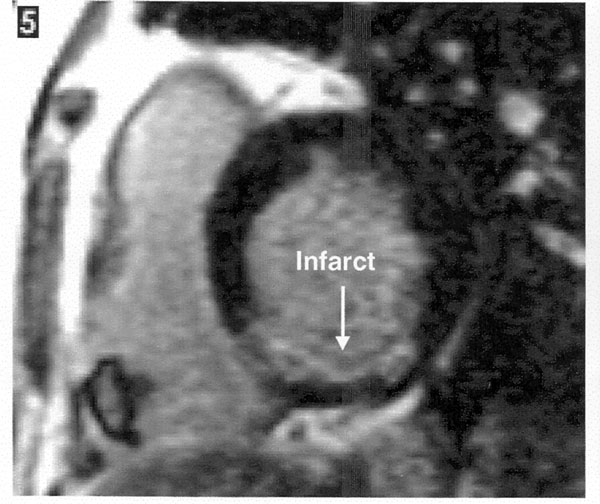
**Cardiac magnetic resonance imaging showing the subendocardial infarction of the right ventricle**.

The patient was commenced on intravenous heparin. Thrombolysis was considered but not given as he had stabilized and the mass was not mobile on ECHO. There was also concern regarding disintegration of the thrombus after thrombolysis. Repeat ECHO after 1 week showed no difference in the size of the mass. As the RV function was normal on the ECHO and the mass showed no change in size, it was decided to evaluate the mass further with cardiac magnetic resonance imaging (MRI). This revealed a mildly dilated RV, a hypokinetic inferior wall and a RV ejection fraction at the lower limit of normal. A mural thrombus was identified attached to the base of the RV. There was also evidence of recent subendocardial infarction of the RV. The conclusion was of a recent infarction of the inferior wall of the RV with secondary mural thrombus formation (Figure 3).

Coronary angiography was subsequently performed that demonstrated an occlusion of the right coronary artery at the ostium, with no angioplasty option. The patient is currently on warfarin and continues to feel well and remains asymptomatic.

## Discussion

Premature coronary artery disease is relatively rare. Most young patients do not experience angina and tend to present differently to older patients with ischaemic heart disease [1]. Further isolated infarction of the right ventricle, as in this patient, is extremely rare [2], even in older patients.

Having received several courses of antimicrobial therapy without effect, despite the lack of demonstrable risk factors, pulmonary embolus had to be considered high on the list of differential diagnosis for this patient presenting with dyspnoea, pleuritic chest pain and haemoptysis. A significantly elevated D-dimer and evidence of right heart strain with tachycardia on ECG added further impetus to this diagnosis. At presentation, there were no symptoms or signs to indicate ischaemic heart disease, with cigarette smoking being the only obvious risk factor. He also denied any recent travel or contact with tuberculosis.

Imaging subsequently confirmed the presence of pulmonary emboli and a RV mural thrombus. A review of the literature confirmed this to be an unusual case. Simultaneous diagnosis of vascular occlusion in both the lung and coronary arteries with an associated mural thrombus in a previously fit man of this age is extremely uncommon.

Hypercoagulable states such as nephrotic syndrome [3,4] and antiphospholipid syndrome [5] are associated with pulmonary embolus and myocardial infarction in individuals under 40 years of age. Cases of pulmonary and cardiac disease have also been reported in individuals diagnosed with Behçet's disease [6] with at least one report of a patient with Behçet's disease simultaneously being diagnosed with RV thrombus and pulmonary embolism (PE) [7]. However, our patient had no family or personal history of venous or arterial occlusion. He had no signs or symptoms suggestive of nephrotic syndrome - his serum albumin was within normal limits and he had no signs of peripheral oedema. He also demonstrated no symptoms of oral or genital ulceration, skin lesions or eye pathology as would be expected in Behçet's disease. The patient denied taking any recent prescribed or self-administered medications that may have caused a hypercoagulable state. Assays for hypercoagulable states were planned to be carried out after completion of treatment with warfarin.

Since no causative underlying pathology was identified, it remains likely that this is an unusual case of a premature myocardial infarction in a patient under 40 years of age presenting primarily with secondary complications. Yet, as stated, other than smoking, this patient lacked risk factors for myocardial infarction expected in an individual of his age. He had no family history of vascular events at a young age. A subsequent glucose tolerance test did not demonstrate diabetes mellitus. His lipid levels were found to be in the normal range and he was not hypertensive at any stage throughout his admission.

This case illustrates the benefits of combined information gained from different imaging modalities when appropriate. The combined information from the modalities of CTPA, echocardiography, cardiac MRI and coronary angiography confirmed the presence of PE and identified the underlying cause as a right coronary artery occlusion.

## Conclusions

This is an unusual case of a premature myocardial infarction, presenting primarily with secondary complications.

## Abbreviations

CT: computed tomography; CTPA: computed tomography pulmonary angiogram; ECG: electrocardiogram; ECHO: echocardiogram; MRI: magnetic resonance imaging; PE: pulmonary embolism; RV: right ventricle.

## Consent

Written informed consent was obtained from the patient for publication of this case report and any accompanying images. A copy of the written consent is available for review by the Editor-in-Chief of this journal.

## Competing interests

The authors declare that they have no competing interests.

## Authors' contributions

SG and MG contributed to writing and editing the case report. RG carried out the literature search while MA and AS performed the coronary angiography. MG obtained the pictures. All the authors read and approved the final manuscript.
